# Prostate response to prolactin in sexually active male rats

**DOI:** 10.1186/1477-7827-4-28

**Published:** 2006-05-17

**Authors:** Maria Elena Hernandez, Abraham Soto-Cid, Fausto Rojas, Luz I Pascual, Gonzalo E Aranda-Abreu, Rebeca Toledo, Luis I Garcia, Andres Quintanar-Stephano, Jorge Manzo

**Affiliations:** 1 Instituto de Neuroetologia, Universidad Veracruzana, AP 566, Xalapa, Ver., 91000, Mexico; 2 Facultad de Quimica Farmaceutica Biologica, Universidad Veracruzana, Xalapa, Ver., 91000, Mexico; 3 Centro de Ciencias Basicas, Universidad Autonoma de Aguascalientes, Aguascalientes, Ags., 20100, Mexico

## Abstract

**Background:**

The prostate is a key gland in the sexual physiology of male mammals. Its sensitivity to steroid hormones is widely known, but its response to prolactin is still poorly known. Previous studies have shown a correlation between sexual behaviour, prolactin release and prostate physiology. Thus, here we used the sexual behaviour of male rats as a model for studying this correlation. Hence, we developed experimental paradigms to determine the influence of prolactin on sexual behaviour and prostate organization of male rats.

**Methods:**

In addition to sexual behaviour recordings, we developed the ELISA procedure to quantify the serum level of prolactin, and the hematoxilin-eosin technique for analysis of the histological organization of the prostate. Also, different experimental manipulations were carried out; they included pituitary grafts, and haloperidol and ovine prolactin treatments. Data were analyzed with a One way ANOVA followed by post hoc Dunnet test if required.

**Results:**

Data showed that male prolactin has a basal level with two peaks at the light-dark-light transitions. Consecutive ejaculations increased serum prolactin after the first ejaculation, which reached the highest level after the second, and started to decrease after the third ejaculation. These normal levels of prolactin did not induce any change at the prostate tissue. However, treatments for constant elevations of serum prolactin decreased sexual potency and increased the weight of the gland, the alveoli area and the epithelial cell height. Treatments for transient elevation of serum prolactin did not affect the sexual behaviour of males, but triggered these significant effects mainly at the ventral prostate.

**Conclusion:**

The prostate is a sexual gland that responds to prolactin. Mating-induced prolactin release is required during sexual encounters to activate the epithelial cells in the gland. Here we saw a precise mechanism controlling the release of prolactin during ejaculations that avoid the detrimental effects produced by constant levels. However, we showed that minor elevations of prolactin which do not affect the sexual behaviour of males, produced significant changes at the prostate epithelium that could account for triggering the development of hyperplasia or cancer. Thus, it is suggested that minute elevations of serum prolactin in healthy subjects are at the etiology of prostate abnormal growth.

## Background

The prostate is a key gland in the sexual physiology of male mammals.

Its location in the reproductive tract influences several vital functions as those related to micturition, seminal emission and ejaculation. In the rat, it is wrapping the proximal region of the urethra, known as the prostatic urethra, with a size that equals the empty bladder. The prostate is a globular gland organized in two regions, the dorsolateral or dorsal prostate and the bilobulated ventral prostate. The histological observation of the gland reveals that in the two regions it is organized in several alveoli surrounded by the stroma area. Each alveolus is arranged with a secretory epithelium that is in charge of the synthesis of prostatic secretions, which are necessary for fertility. The main type of epithelial cells has a columnar shape and is the target for the endocrine stimulation of the gland.

Steroid hormones have a strong influence on the physiology of the prostate. Androgen stimulation produces several changes in the gland with differential effects on the dorsal or ventral regions [1,2], and castration reduces significantly the weight of the gland and alters the organization of epithelial cells [3]. Among androgens,	it has been shown that the 5α-dihydrotestosterone is more potent than testosterone to maintain some characteristics of the prostate [4], but also is known that estrogens are important for the maintenance of some functions of the gland [5]. On the other hand, peptide hormones have received considerably less attention regarding their role in prostate function. However, it has been shown that prolactin (PRL) has a biphasic influence on prostatic organization [6], and that the prostate shows the expression of mRNA for PRL receptors [7]. Thus, in this work we were interested in further investigate the effects of PRL on the organization of the prostate in sexually active male rats.

PRL is a hormone synthesized and secreted into the blood by lactotrophs in the adenohypophysis. It participates in different functions, of which those relating to reproduction have been studied the most. As is the case with other hormones produced in the hypophysis, PRL displays a variable serum level during the day. In adult rats it has been shown that PRL peaks around the light-dark transition period [8,9]. Most studies of PRL in males have focused on an analysis of its abnormally high levels, known as hyperprolactinemia (hyperPRL). Subjects with hyperPRL show a severe effect in the potency for penile erection [10,11]. In the behavioural context, hyperPRL males can initiate copulation but either do not ejaculate or show a significant reduction in their ejaculatory potential [12,13]. Hence, hyperPRL males generally take a longer time to ejaculate than control males [12-15], with a concurrent higher number of mounts and lower number of intromissions [12,13,15-17]. The time course effect of hyperPRL on sexual behaviour seems to be around 10 to 15 days [14,15]. Notwithstanding these findings, every study confirms that a constant, high level of PRL reduces the sexual function of males. In contrast, a transient elevation of endogenous PRL is observed during the sexual behaviour of male rats or in males attempting to copulate with nonestrous females, and the execution of the behaviour is necessary since the odor alone from estrous females is unable to cause this increase [18]. Additionally, an acute or chronic treatment to induce an increase of PRL in male rats results in a respective stimulatory or inhibitory modification of sexual behaviour [19]. This means that in contrast to constant conditions, a brief surge of PRL seems necessary for a proper expression of sexual function in males.

In view of this information, it is clear that there is a correlation between sexual behaviour, PRL release and prostate physiology. The correlation seems unidirectional since sexual activity influences prostatic function, but the lack of the prostate does not alter the behavioural context [20]. Thus, here we used the sexual behaviour of male rats as a model for studying this correlation. This behaviour in rats has a well-known stereotyped pattern. Males display several mounts and intromissions before ejaculation, and all these parameters occur recurrently in such a way that a male can achieve several ejaculatory series in a single sexual encounter. Taking advantage of this behavioural pattern, we developed experimental paradigms to determine, in a longitudinal study, the influence of sexual behaviour on the level of PRL and prostate organization in male rats displaying up to four consecutive ejaculations.

## Methods

### Subjects and housing

Sexually experienced Wistar male rats were used (250–300 g/bw). Males were living in a room with an inverted light-dark cycle (12–12 hr, lights off at 0800 hrs). Every rat was manipulated daily, hence they were habituated to experimenter contact and to the environment in order to avoid the stress-induced PRL release. Ovariectomized females were used, and their sexual receptivity was induced with steroids, dissolved in sesame oil. Accordingly, subcutaneous injections of estradiol benzoate (10 μg) and then progesterone (2 mg) were administered 48 and 4 hrs before tests, respectively. Rats were housed in plastic cages (50 × 30 × 20 cm) containing wood chip bedding and rooms were under a controlled temperature (22 ± 2°C), with food (Harlan Mexico rodent chow) and water available *ad libitum*. Every surgical intervention and manipulation of rats was guided by the Society for Neuroscience Policy on the Use of Animals in Neuroscience Research.

### Experiment 1

This experiment was designed to obtain the baseline levels of serum PRL in the dark phase. Four groups were formed: G8 (n = 10), G12 (n = 12), G16 (n = 12), and G20 (n = 12). The number indicates the hour of the day on which blood was collected, such that the term 'G12' would indicate the sample was taken at 12:00 hrs. Males were cannulated for blood collection (see below) two days before the beginning of the experiment. Blood samples (0.2 ml) were collected once per animal on the hour indicated by the group, but samples at 08:00 hrs were taken about 5 minutes after the designated hour, and samples at 20:00 hrs were taken about 5 minutes before the designated hour.

Males were cannulated for blood collection. They were anesthetized with sodium pentobarbital (Smith Kline, Mexico; 30 mg/kg bw, i.p.), and the right jugular vein was exposed. The vein was half-cut and 2.5 cm of a cannula (Dow Corning, USA, 0.30 mm ID and 0.64 mm OD) introduced to leave the tip in the right atrium. The cannula and vein were fixed and a small loop of the cannula was positioned in the ventral neck area to avoid displacement during head movements. The other side of the cannula was guided subcutaneously to the back and exposed through a hole made in the skin. The skin in the neck was sutured, covering the cannula and loop. Therefore, only the end of the cannula in the back was exposed. It was filled with saline solution with a Hamilton syringe, the tip was closed with a small metal plug, and it was covered with a belt (1.5 cm wide) that surrounded the thoracic area of the body. The belt was lab-made for this experiment and was designed for two purposes: to protect the cannula from male scratching movements, and to allow the male to copulate without any interference. Cannulated males were placed alone in a cage. To collect blood, a small drop of a heparin solution was placed in a Hamilton syringe. The syringe was connected to the tip of the cannula and blood extracted and mixed inside the syringe with the heparin solution. After blood collection, a second Hamilton syringe was connected to the cannula to inject 0.2 ml of saline solution to wash most of the blood left inside the cannula and to avoid coagulated plugs. Blood samples were centrifuged, and the plasma obtained was stored at -20°C until assayed.

PRL levels in plasma samples were measured using the ELISA procedure. A 96 well plate was coated with PRL (NIDDK-rPRL-I-6) at a 10 ng/50 μl concentration in carbonate buffer (sodium carbonate 0.1 M, and sodium bicarbonate 0.035 M), and incubated at 4°C for 24 hr. On the other hand, using eppendorf tubes, PRL standard curves were done in triplicate at several concentrations in PBS 0.1 M and Tween 20 0.05% (concentrations: 120, 60, 30, 25 and 7.5 ng/ml). Also, both diluted plasma samples (1:6) and standard curves were incubated in parallel with the first antibody (1/100, NIDDK-anti-rPRL-RP-3) overnight at 4°C. Then, the plate was washed (3 times, 5 min each) with PBS-Tween 20 solution, followed by the adding of bovine serum albumin 1% for an hour, and washed once for 3 min with the PBS-Tween 20 solution. 50 μl of standard curve and plasma sample were took, added to the wells, and incubated at 4°C for 24 hr. Again, the plate was washed (3 times, 3 min each) with PBS-Tween 20 solution, incubated with the second peroxidase-conjugated antibody (1/800) at room temperature for 2 hr, and washed (3 times, 5 min each) with PBS-Tween 20 solution. The plate was developed with orthophenildiamine (10 mg) and hydrogen peroxide (3%) in citrate buffer (citric acid 0.05 M, and dibasic sodium phosphate 0.1 M) for 10 min, and stopped with cold sulphuric acid (4 N). The plate was read at 490 nm. The sensitivity was about 3 ng/ml, and intra and interassay coefficient variations were 9.2 and 15.5% respectively.

### Experiment 2

The experimental paradigm of this study was used to determine the effect of consecutive ejaculations (up to 4) on the level of serum PRL and on the histology of the ventral and dorsal prostate. Males were prepared for blood collection two days before the beginning of tests, and females were treated appropriately to induce a state of sexual receptivity. Experiments were carried out under red light between 12:00 and 16:00 hrs, the time considered ideal for the proper execution of sexual behaviour in rats [21]. A male rat was taken from its cage and a blood sample obtained (0.2 ml). It was then placed in a Plexiglas arena (60 cm diameter × 60 cm high), and a receptive female was introduced five minutes later. Parameters detailing the sexual behaviour of the male were recorded immediately [22], but only the Hit Rate parameter is showed in results for the last experiment; it is a proportion obtained after the computation of the number of intromissions divided by the sum of the number of intromissions and the number of mounts [23]. This is a good indicator of the appropriate execution of male's sexual behaviour.

The number of consecutive ejaculations marked the assigned group of the animal. Males from group E1 (n = 17) were allowed to ejaculate once, and then a post-ejaculatory blood sample was obtained. Group E2 (n = 13) males were allowed to ejaculate twice, group E3 (n = 12) males were allowed three ejaculations, and group E4 (n = 12) males were allowed four ejaculations, with post-ejaculatory blood samples being obtained after the final ejaculatory event of each male. Every animal was thus sampled twice per test: once before and once after its ejaculatory series, and samples analyzed with the ELISA procedure. On the other hand, about 50% of males in each group were killed after its respective ejaculation, the prostate obtained, weighed and prepared for histology with the hematoxilin-eosin stain.

The stain with hematoxilin-eosin started by immersing the prostate for 24 h in Helly fixative (Mercuric Chloride 5%, Potassium Dichromate 2.5%, Sodium Sulfate 1%, and Formalin 37%); then the fixative was washed with tap water for 30 min. Dehydration was carried out by immersing the prostate in ethanol 70% (1 hr), 80% (1 hr), 96% (3 times, 2 hrs each), absolute ethanol (overnight), and absolute ethanol (2 times, 1 hr each). Clearing was done by immersing the prostate in xylene (3 times, 1 hr each). Both dehydration and clearing were carried out under continuous shaking. Then, the prostate was embedded in melted paraffin (2 times, 2 hr each at 57°C) and a block was done by using a microtome cassette. The paraffin block was sectioned on a Leica microtome (5 μm). Sections were placed on a mounting bath with gelatinized water at 52°C and tissues mounted on slides that were placed for 1 hr in an oven (~58°C). For staining, tissues were immersed in xylene (3 times, 5 min each), absolute ethanol/xylene 1:1 (5 min), ethanol 96% (3 min), iodine alcohol (5 min), sodium thiosulfate (10 min), tap water (2 min), Mayer's Hematoxilin (10 min), tap water (30 sec), acid ethanol (fast bath), tap water (10 sec), lithium carbonate (30 sec), tap water (10 sec), Eosin Y (4 fast baths), ethanol 96% (3 min), absolute ethanol (2 min), absolute ethanol/xylene 1:1 (2 min), and xylene (5 min). Finally, sections were coverslipped with undiluted Permount. Each slide had sections from just one region of the prostate, i.e., we obtained ventral slides and dorsolateral slides. The slides were observed in an Olympus Provis AX-70 microscope, and images obtained and analyzed with the Image-Pro Plus software. Measures obtained were the height of epithelial cells and the area of the alveoli.

### Experiment 3

This experiment was done to determine the effect on the prostate of treatments that are known to increase PRL serum level and decrease sexual behaviour. Three groups of males were used (n = 6 each): control males (without treatment); males treated with haloperidol; and males with a pituitary graft under the kidney capsule. After 15 days of treatment, males were killed, the prostate obtained, weighed and prepared for histology with the hematoxilin-eosin stain. Blood samples were also obtained from these males to get the profile of PRL in serum. Haloperidol was administered with implants that consisted of silastic capsules (10 mm long, 1.58 mm i.d., 3.06 mm o.d.) filled with the crystalline drug (~9 mg). The capsule was placed subcutaneously in the nape of the neck under ether anaesthesia. Pituitary grafts were obtained from a donor male that was killed immediately after pituitary removal.

### Experiment 4

In this experiment, a controlled administration of exogenous PRL was used to determine the effect on the prostate of sexual behaving males. Three groups of males were used (n = 30 each): Intacts with no treatment, Vehicle injected and PRL injected. Treated males were injected subcutaneously with two daily doses (10:00 and 18:00 hrs) of vehicle or ovine PRL. On day 3 after the beginning of treatments, every male was tested for sexual behaviour. Then, 6 males of each group were killed, the prostate obtained, weighed and prepared for histology with the hematoxilin-eosin stain. Blood samples were also obtained from these males to get the profile of PRL in serum. Remaining males were returned to the animal room and the same procedure repeated on days 6, 9, 12 and 15.

Ovine PRL was obtained in bottles of 33.3 μg (Sigma Chemical, Mexico) that were diluted in injectable grade water (14.5 ml) to obtain a concentration of 100 μM. 100 μl of this stock solution was diluted (1:4) in the injectable water. 100 μl of this final solution were in each dose injected to experimental males (~50 μg of PRL per dose).

### Statistics

Nonparametric statistics were used in Experiments 1 and 2. Between-group comparisons of data from Experiment 1 were made with Kruskal-Wallis (KW) analysis of variance, and paired contrasts were obtained with Mann-Whitney (MW) tests. PRL data from Experiment 2, comparing pre-and post-ejaculatory results of correlated groups, were analyzed with the Wilcoxon Signed-Rank test. Experiment 3 and 4 were analyzed with a One Way ANOVA followed by the post hoc Dunnet test when the F value showed significant differences at p < 0.05. In every statistics, significant differences were inferred when p < 0.05.

## Results

### Experiment 1

**Figure 1:**
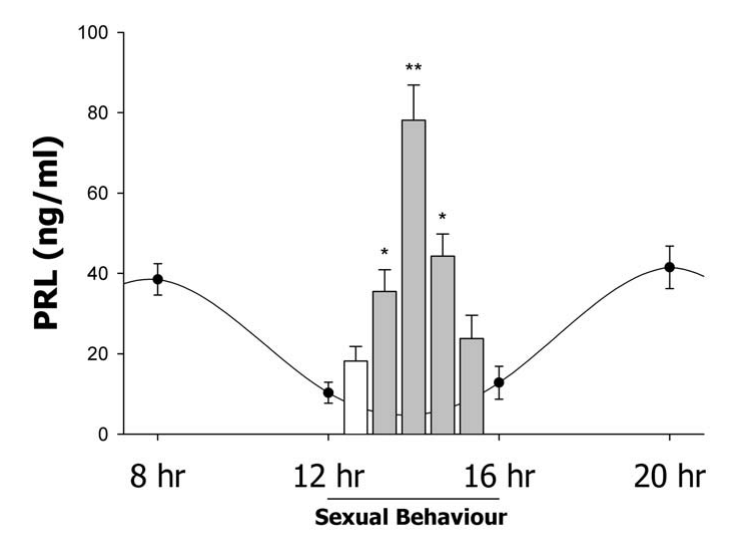
**Serum levels of PRL**. The graph represents the comparison of basal values (sine wave) and the values produced after consecutive ejaculations between 12 and 16 hrs (bars). Significant changes are shown just for bars. The white bar is a representative value of the level of PRL obtained before sexual behaviour. Gray bars represent the values obtained after each of the four consecutive ejaculations (mean ± SEM). Asterisks represent significant changes (* = p < 0.05, ** = p < 0.01) in comparison to the paired white bar of each group. The mating-induced PRL is transient and reached higher values after the second ejaculation; ng/ml = nanograms per millilitre.

PRL was detected in every plasma sample analyzed. However, the concentration was different depending on the hour of the day in which the sample was taken. Statistical analysis showed that PRL had overall differences, and MW tests revealed significant differences between the samples at 12:00 and 16:00 hrs and those taken at the transition between the dark-light and light-dark phases. A consideration of all the data reveals that the lowest PRL concentration (~12 ng/ml) represents the baseline of the hormone, with two significant peaks being evident at the dark-light-dark transitions which increased the PRL concentration to ~38 ng/ml (Fig. 1).

### Experiment 2

PRL levels showed a brief but significant increase after consecutive ejaculations. As expected, a quantity of PRL was detected before the male was introduced to the sexual behaviour arena, with this concentration ~18 ng/ml being similar to the baseline obtained in Experiment 1. The concentration of serum PRL was significantly higher in samples taken after the first, second and third ejaculations in comparison to its paired precopulatory value, but after the second ejaculation the level of PRL reached its maximum. A Gauss-like curve of PRL was therefore detected (Fig. 1). It started to rise after the first ejaculation (~35 ng/ml, p < 0.05), formed a peak after the second ejaculation (~80 ng/ml, p < 0.01), started to decrease after the third ejaculation though still remaining high (~45 ng/ml, p < 0.05), and after the fourth ejaculation the PRL concentration returned to baseline levels. Placing this data on the curve of PRL quantified between 12 and 16 hr of Experiment 1 reveals that sexual behaviour causes a temporary and significant increase of PRL release, even to a level that is higher than the peak observed in the dark-light-dark transitions (Fig. 1).

Histological observations of the prostate showed that consecutive ejaculations did not produce any change on the tissue (data not shown). Every data regarding control males in the next two experiments are equivalent to the tissue parameters observed here.

### Experiment 3

**Figure 2:**
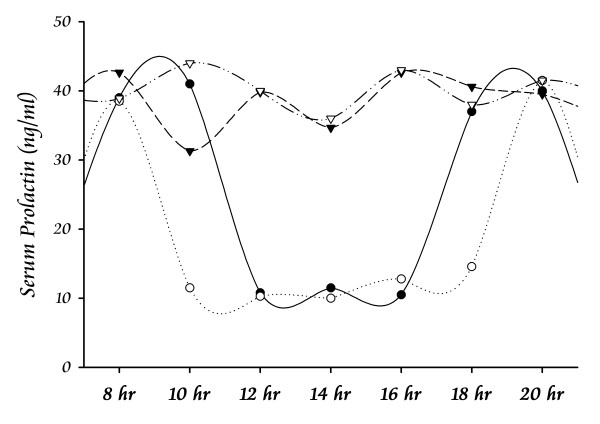
**Serum levels of PRL after Treatments**. The graph represents the estimated daily profile of PRL in serum after experimental treatments. Lines show the comparison between basal values (◯) and the values obtained after treatments with haloperidol (▼), pituitary grafts transplants (▽), and ovine PRL administration (●). Thus, the experimental paradigms allowed continuous levels and transient levels. Each point represents the mean value. The profile induced by sexual behaviour is not represented (see Fig. 1).

**Figure 3:**
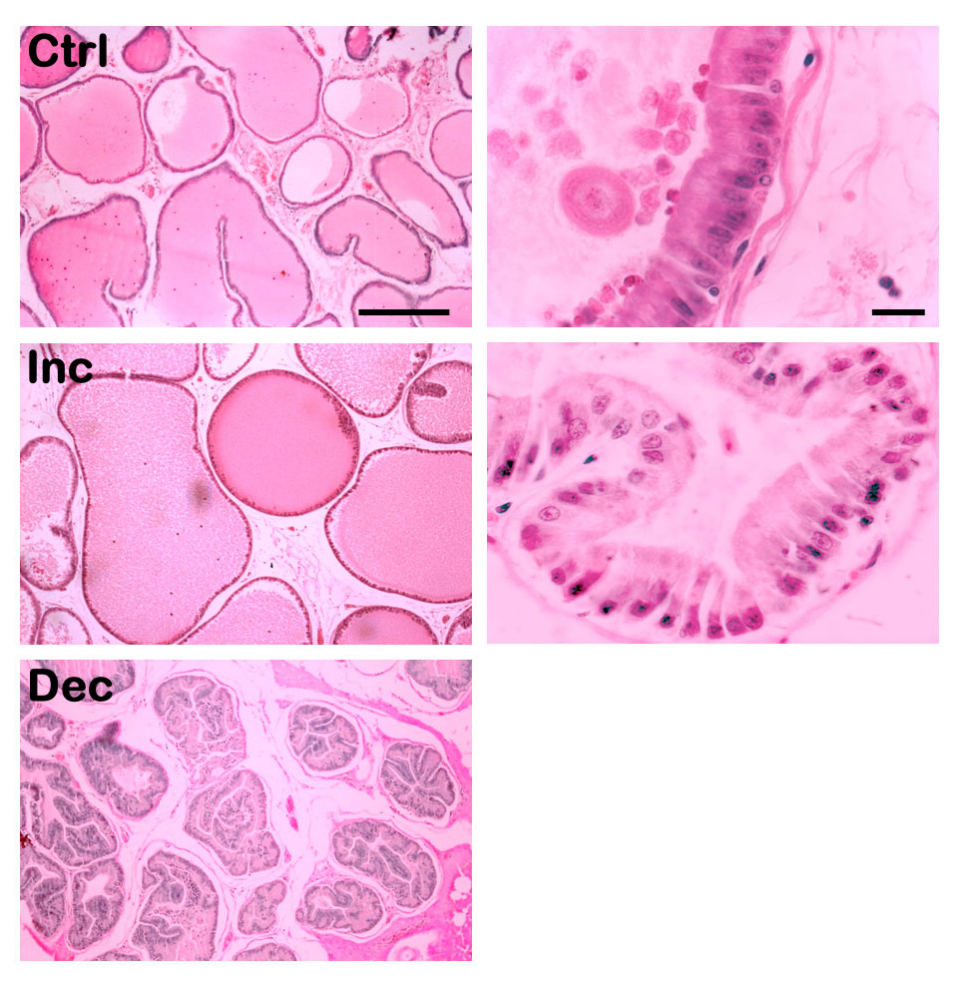
**Histology of the Ventral Prostate**. Examples of images obtained after the different treatments. The left column represents the effect on alveoli, and the right column the effect on the epithelium. The top row shows the histology in intact (Ctrl) subjects, while the middle shows increments (Inc; from Experiment 3, see Fig. 5) and the bottom decrements (Dec; from Experiment 4, see Fig. 6) of the parameters. A decrement in the epithelium was never observed, so there is not a picture to show. Left scale bar = 250 μm; Right scale bar = 10 μm.

**Figure 4:**
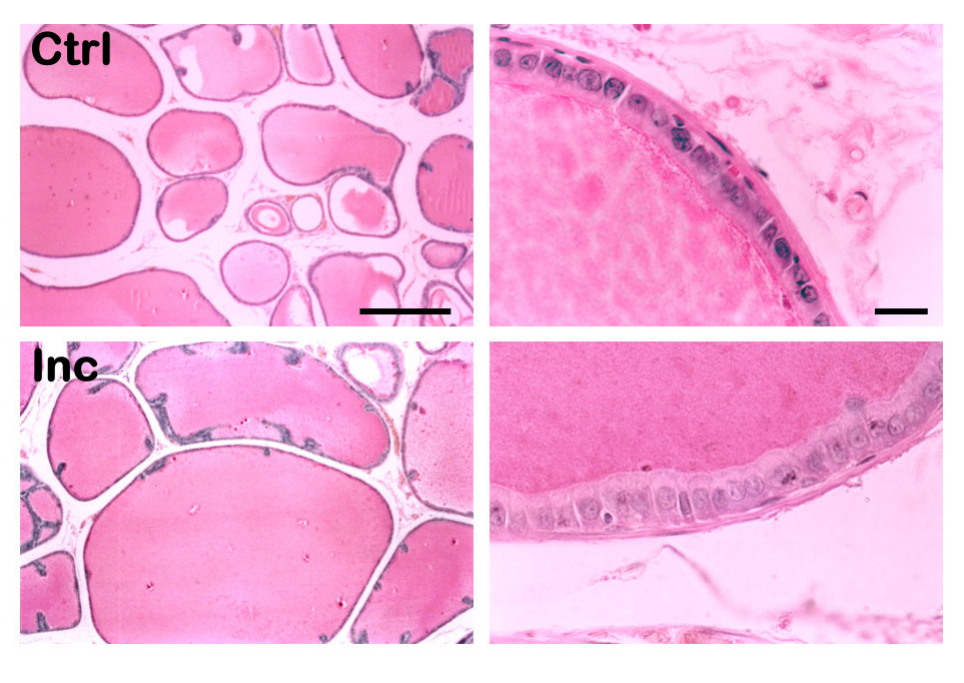
**Histology of the Dorsal Prostate**. Examples of images obtained after the different treatments. The left column represents the effect on alveoli, and the right column the effect on the epithelium. The top row shows the histology in intact (Ctrl) subjects, while the bottom shows increments (Inc) of the alveoli area (from Experiment 3, see Fig. 5) and epithelial height (from Experiment 4, see Fig. 6). Neither decrement in the alveoli nor in the epithelium was observed, so there is not a picture to show. Left scale bar = 250 μm; Right scale bar = 10 μm.

**Figure 5:**
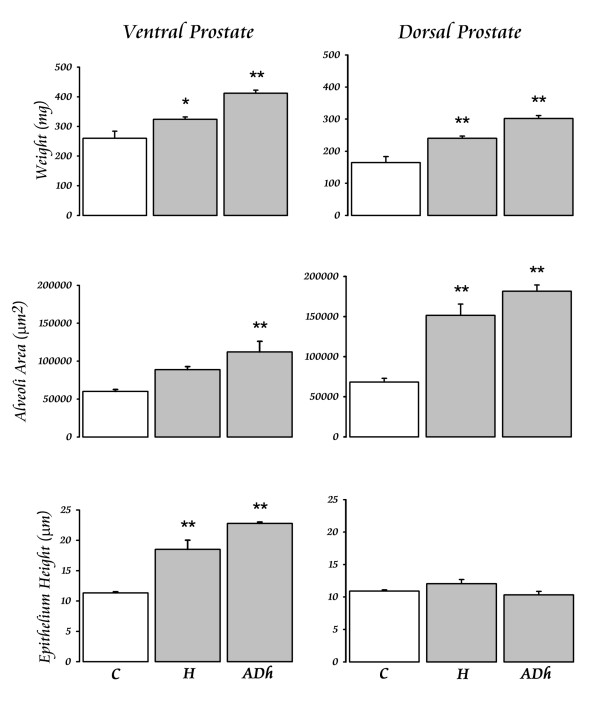
**Prostate responses to constant levels of PRL**. The increase of serum PRL after treatment with haloperidol (H) or after an adenohypophysis graft under the kidney capsule (Adh) produced increments in the three measured parameters. However, the ventral and dorsal regions of the prostate exhibited some differences, i.e., H did not modify the alveoli area at the ventral prostate, and both Adh and H did not modify the epithelium height at the dorsal prostate. Gray bars are compared vs. the white bar representing the control (C) value. * = p < 0.05; ** = p < 0.01.

The continuous elevation of serum PRL (Fig. 2) by administration of haloperidol (Hal) or adenohypophysis (Adh) transplant produced significant effects on the prostate tissue (Fig. 3 and 4). The weight of the gland increased after both treatments, but the Adh produced a higher increase in the ventral prostate (Fig. 5). The alveoli area increased significantly in both regions of the prostate after Adh; however, Hal produced an increase only at the dorsal region without affecting the ventral prostate (Fig. 5). The epithelial cells height increased significantly in the ventral prostate after Adh or Hal, but there was not any change at the dorsal region (Fig. 5).

### Experiment 4

**Figure 6:**
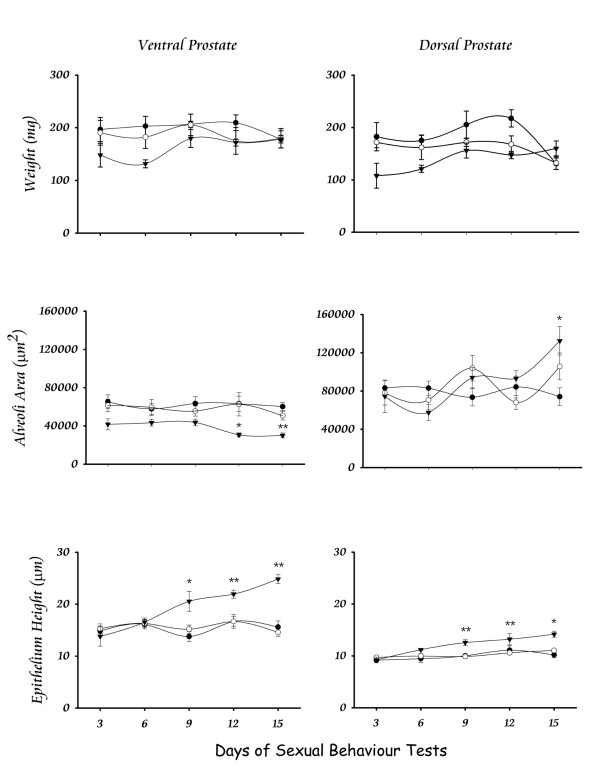
**Prostate responses to a small dose of PRL insexually active males**. The treatment with daily doses of ovine PRL (▼) in sexually active males did not modify the weight of the prostate. However, a decrement was observed in the alveoli area of the ventral prostate, and also it was observed a reliable increase of this parameter in the dorsal prostate after 15 days of treatment or fifth behavioural test. The epithelium height was increased starting at the day 9 or third behavioural tests in both regions of the prostate. The incremental slope was constant with a higher magnitude at the ventral prostate. No modification was observed in untreated (◯) or vehicle treated (●) males. * = p < 0.05; ** = p < 0.01.

**Figure 7:**
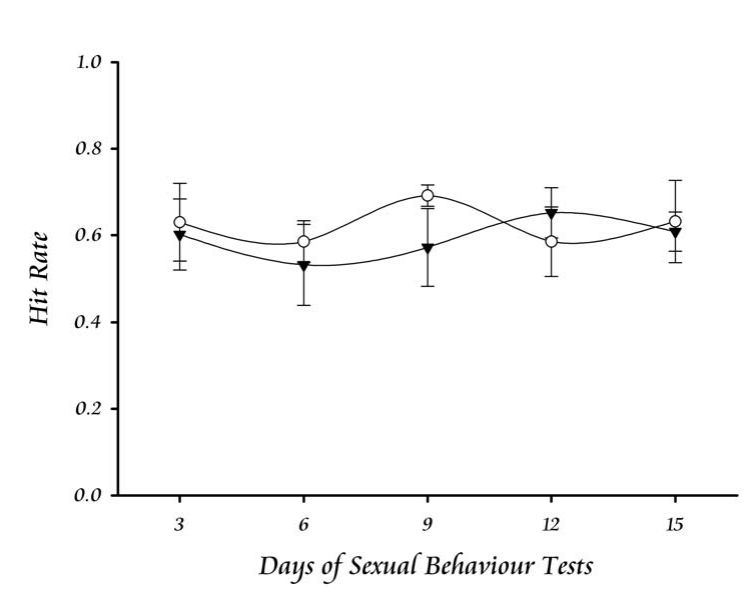
**Sexual behaviour of male rats treated with a small dose of PRL**. The treatment with daily doses of ovine PRL (▼) in sexually active males did not affect the potency for sexual behaviour. The proportion showed by the hit rate parameter is considered a good indicator of sexual behaviour. Treated males had similar parameters than controls (◯).

The exogenous controlled-administration of PRL produced transient elevations of serum PRL (Fig. 2) and significant effects on the prostate tissue without affecting the sexual behaviour of males. The weight of the gland remained constant in both the ventral and dorsal regions (Fig. 6). However, the alveoli area at the ventral region showed a significant decrease after 12 days; the opposite effect was observed for the dorsal region, but a significant value was only seen after 15 days of treatment (Fig. 6). The epithelial cells height increased significantly after 9 days of treatment in both the ventral and dorsal prostate (Fig. 6). As an indicator that the sexual behaviour was not affected, Fig. 7 shows the Hit Rate parameter.

## Discussion

Our data show that male PRL has a basal level with two peaks at the light-dark-light transitions. These findings support the work of other researchers who have reported one or two peaks under different experimental conditions [8,24]. The fluctuation of PRL levels could be useful for a number of bodily functions. It has been shown that PRL serves to potentiate the effect of androgens on the accessory glands and on the increase of luteinizing hormone receptors in Leydig cells, that in turn increases testosterone synthesis [25]. Maintenance of the testis therefore seems to be a key function of PRL, and here we showed that PRL is also important for the physiology of the prostate.

Consecutive ejaculations in the same day have a strong effect on the transient release of PRL in serum. A significant increase of PRL was observed after the first ejaculation, and reached the highest level after the second ejaculation. The level of PRL then started to decrease. After the third ejaculation it was still significantly elevated, and by the fourth ejaculation it returned to pre-copulatory levels. A question arising from these results focuses on determining the parameter of sexual behaviour that is triggering the release of PRL. Attempts by males to copulate with nonestrous females (mounting, without intromissions) stimulate PRL release [26], and here we also showed that the serum level of PRL fluctuates in a specific pattern after one or more ejaculations. Thus, it seems that the values we recorded are the sum of individual influences of mounts, intromissions and ejaculations. However, further studies are needed to determine the extent of the influence of each copulatory parameter in this process. A remarkable observation was the fact that the second copulatory series triggered the maximal concentration of PRL in serum. Considering that the third and fourth ejaculatory series were unable to produce a higher elevation of PRL, we would like to suggest a mechanism to account for these observations. The first copulatory series primes the system for a maximal response after the second ejaculatory series. The highest PRL level observed after the second ejaculation then triggers a long refractory period that makes the system controlling the release of PRL unresponsive to the influence of further mounts, intromissions and ejaculations. As a consequence, PRL starts to decrease (post-third ejaculation value) until it reaches its baseline level (post-fourth ejaculation value).

PRL has an important influence on male reproduction by modulating the histological structure of the prostate. It is known that PRL receptors are expressed in the epididymis, seminal vesicle and prostate [7]. Consequently, the increase of PRL during the first and second copulatory series could serve to trigger the synthesis and release of the contents of these glands in order to have a sufficient quantity of semen to expel in each ejaculation. Here we showed that the chronic increase of PRL, by Hal or Adh transplant, had significant effects on the prostate, although Adh transplant produced more effects than Hal treatment. The weight of the gland and the alveolar area increased significantly, and the epithelial height also increased but only at the ventral region of the prostate. Thus, Adh transplants and Hal treatments produced similar effects on the prostate after inducing high and continuous level of serum PRL, similar to other reports too [27,28]. The mechanism of PRL to induce these changes is unknown, but as described by other authors [29-31], this hormone has a number of effects at the prostate tissue as those related to citrate production, apoptosis decrease by overexpression of Bcl-2, or synergistic effects with androgens. Accordingly, we hypothesize that the observed growth of different histological parameters of the gland is related to an increased synthesis and release of its contents to the alveoli area. Whether the content is citrate or another compound is a topic that still deserves further research. Furthermore, as widely described, our work also showed that the prostate is a complex gland, since each lobule seems to have its own molecular characteristics responding differently to the same treatment.

Our final experiment showed that the periodic execution of sexual behaviour, producing transient elevations of serum PRL, do not induce any change in the prostate tissue. However, the exogenous administration of ovine PRL at a dose that does not affect copula parameters showed that the weight of the gland did not change, the alveolar area decreased with time only at the ventral region, and the epithelial height increased in the ventral and dorsal regions but with a higher magnitude at the ventral prostate. Hence, it is clear that the prostate tissue is capable to function properly during continuous copulation without changing its structure, but after a slight increase in serum PRL the gland has reactions mainly at the epithelium. Epithelial cells response could be due to an increased expulsion of semen or to a decreased potency of the cell to synthesize its product, as suggested by the area decrease of alveoli. These suggestions are supported by other reports showing that PRL in the prostate epithelial cells modifies the organelles in charge of protein synthesis and secretion [32], that PRL produces an increased number of prostate androgen receptor [33], and that PRL along with testosterone modulates the activity of enzymes associated with citrate production [34]. Hence, a number of changes are observed at the epithelial cells in response to a slight increase of PRL. Here, we showed that PRL has a transient elevation after consecutive ejaculations in the same sexual encounter, and that a mechanism exists to inhibit further increase after a third ejaculation. This inhibition could account for a protection of tissues, like the prostate, to maintain its normal organization and function. Thus, our set of experimental paradigms showed that the prostate tissue is highly sensitive to minute elevations of serum PRL beyond the normal levels, which does not affect the health or behaviour of the subject. Our suggestion then, is that this minute elevation of PRL could be at the etiology of prostate hyperplasia and/or cancer. It is known, in humans, that hyperprolactinemia is frequent in both breast and prostate cancer [35,36]. Then, it is suggested that a serum PRL test could be useful to predict the beginning of prostate abnormal growing, even before the prostate-specific antigen appears in serum and much before prostate cancer symptoms occur.

## Conclusion

The prostate is a sexual gland that responds to prolactin. Mating-induced prolactin release is required during sexual encounters to activate the epithelial cells in the gland. Here we saw a precise mechanism controlling the release of prolactin during ejaculations that avoid the detrimental effects produced by constant levels. However, we showed that minor elevations of prolactin which do not affect the sexual behaviour of males, produced significant changes at the prostate epithelium that could account for triggering the development of hyperplasia or cancer. Thus, it is suggested that minute elevations of serum prolactin in healthy subjects are at the etiology of prostate abnormal growth.

## Competing interests

The author(s) declare that they have no competing interests.

## Authors' contributions

MEH is the author in charge of this research line; her lab is specializing in prostate research, so she designed the project in collaboration with JM that has good experience in projects involving the use of sexual behaviour in male rats; at the end both of them were in charge of organizing this manuscript. ASC, FR, and LIP were in charge of the histological procedures and analyses, and also of the drug treatments. GEAA, RT, and LIG were in charge of the ELISA procedure and sexual behaviour recordings; and AQS was in charge of most surgical procedures.
